# Give your ideas a hand: the role of iconic hand gestures in enhancing divergent creative thinking

**DOI:** 10.1007/s00426-024-01932-1

**Published:** 2024-03-28

**Authors:** Gyulten Hyusein, Tilbe Göksun

**Affiliations:** Department of Psychology, Koç University, Istanbul, Turkey

## Abstract

Hand gestures play an integral role in multimodal language and communication. Even though the self-oriented functions of gestures, such as activating a speaker’s lexicon and maintaining visuospatial imagery, have been emphasized, gestures’ functions in creative thinking are not well-established. In the current study, we investigated the role of iconic gestures in verbal divergent thinking—a creative thinking process related to generating many novel ideas. Based on previous findings, we hypothesized that iconic gesture use would facilitate divergent thinking in young adults, especially those with high mental imagery skills. Participants performed Guildford’s Alternative Uses Task in a gesture-spontaneous and in a gesture-encouraged condition. We measured fluency (number of ideas), originality (uniqueness of ideas), flexibility (number of idea categories), and elaboration (number of details) in divergent thinking. The results showed that producing iconic gestures in the gesture-encouraged condition positively predicted fluency, originality, and elaboration. In the gesture-spontaneous condition, producing iconic gestures also positively predicted elaboration but negatively predicted flexibility. Mental imagery skills did not interact with the effects of gestures on divergent thinking. These results suggest that iconic gestures are a promising candidate for enhancing almost all aspects of divergent thinking. Overall, the current study adds a new dimension to the self-oriented function of iconic gestures, that is, their contribution to creative thinking.

**Supplementary Information:**

The online version contains supplementary material available at 10.1007/s00426-024-01932-1.

## Introduction

Creativity is defined as the formation of a product or an idea that is both novel and useful (Runco & Jaeger, 2012) and mostly differentiated as divergent and convergent creativity (Guilford, 1950; Mednick, 1962). While divergent thinking is defined as the generation of various new and appropriate ideas, convergent thinking is synonymous with having an insight into a problem or finding the best possible answer. Recently, studies have highlighted the valuable role of physical experiences and bodily interactions in creative thinking (for a review see Frith et al., 2019). For example, walking enhanced divergent thinking and the generation of insightful analogies (Oppezzo & Schwartz, 2014), fluid arm movements benefitted convergent thinking (Slepian & Ambady, 2012) and creative fluency (Imaizumi et al., 2020), and enacting metaphors, such as “thinking outside the box,” promoted divergent and convergent thinking in problem-solving (Leung et al., 2012). These findings are in line with the *embodied cognition* paradigm, which states that the way we think is influenced by the way we interact with our physical environment, and our thoughts are distributed throughout our body and physical movements (Lakoff & Johnson, 1980; Stanciu, 2015).

A case of embodied physical movements, that occur spontaneously when one is speaking or thinking and carry mental representations within themselves, is *hand gestures* (Kendon, 2004; McNeill, 1992). The Gesture as Simulated Action (GSA) framework, for example, directly links gestures to the embodiment of the mind (Hostetter & Alibali, 2018), suggesting that gestures reflect the motor activity happening when people think and speak about the mental simulations of perceptual states and motor actions. Hand gestures aid both the speaker and the listener in conveying and grasping the meaning of speech and thus facilitate communication (Clough & Duff, 2020; Kita et al., 2017; Pine et al., 2010). Gestures also support internal cognitive processes, such as facilitating word retrieval, activating mental images, and reducing cognitive load (e.g., Goldin-Meadow, 2001; Kita et al., 2017; Krauss et al., 2000). The research has shown that using gestures while speaking has the potential to benefit two distinct types of creative thinking—convergent (Hyusein & Göksun, 2023) and divergent thinking (Frith, 2020; Kirk & Lewis, 2017; Laurent, 2020; Liao & Wang, 2019) both in children and young adults. However, these effects might depend on additional factors, such as mental imagery skills for convergent thinking (e.g., Hyusein & Göksun, 2023), and individual variability in gesture use for divergent thinking (e.g., Frith, 2020). For example, Hyusein and Göksun (2023) showed that individuals with higher mental imagery skills benefit from gestures when solving convergent thinking tasks while individuals with lower-than-average mental imagery skills do not, and gesturing can even be detrimental to their convergent thinking.

As imagery is an important aspect of both gestures (Hostetter & Alibali, 2007; Kita et al., 2017) and creativity (May et al., 2020; Vellera & Gavard-Perret, 2012, 2016), in the current study, we test an analogous interaction between gestures, mental imagery and divergent thinking. If, as in the case of convergent thinking, gestures positively predict divergent thinking only for high-imagery individuals, the current study can further enlighten us on whether we can enhance creativity through training one’s mental imagery skills and then encouraging them to use their hands to become more creative.

### Embodied creativity

The conventional practices of evaluating creativity usually fail to account for factors other than the implicit cognitive functions that act behind the creative output’s closed doors. It would be erroneous to say there have not been any attempts to give a broader and more holistic definition of creativity. For instance, the 4P framework by Rhodes (1961) suggests that creativity is the interaction of four elements—*person*, *press*, *process*, and *product*. The individual creative characteristics of the *person* when combined with the requirements of the environment (*press*) lead to a *process* of mental operations, the outcome of which is the *product*. Two other *P*s were later added by researchers to make the framework more complete. *Persuasion* (the socio-cultural acceptance of the *product*) was added by Simonton (1990) and *potential* (everybody has the capacity to be creative) by Runco (2003). More recent additions to the *P* frameworks of creativity (e.g., the 8*P*s framework by Sternberg & Karami, 2021) also emphasize that creativity is not merely a personal or cognitive ability but highly dependent on and shaped by the requirements of the environment, the culture, and the dynamics among these. The recent neuroimaging studies also reveal that creativity involves the coordination and collaboration between different networks of the brain, e.g., the cognitive control and the default mode networks (Beaty et al., 2014). Such a coupling of disparate brain regions is a sign that we should move from viewing creativity as a static skill or product and shift our focus to examining it as a more dynamic process.

An essential aspect that could contribute to the dynamic view of creativity as a process is *embodiment* (Frith, 2019; Malinin, 2019). The *embodied cognition* paradigm states that our mental representations are not solely confined to the limits of our brain, but they are distributed throughout the body and intertwined with our bodily movements and physical experiences (Lakoff & Johnson, 1980; Stanciu, 2015). Therefore, our mind works in synchrony with our bodies in the space defined by our environment to shape and create our thoughts, perceptions, and creative ideas. This holistic view has also been incorporated into creativity research with scientists examining creativity as a cognitive process impacted by bodily and social interactions (e.g., Frith, 2019; Glăveanu, 2014; Kimmel & Hristova, 2021; Ross, 2023).

Empirical studies have been extended to test the *embodied creativity account*. Arm movements (Slepian & Ambady, 2012), metaphor enactments (Leung et al., 2012), squeezing objects (Kim, 2015), motor restrictions (Murali & Händel, 2022), body postures (Michinov & Michinov, 2022), embodied creativity training and improvisation (Byrge & Tang, 2015; Felsman et al., 2020) were all related to different measures of creative thinking. For example, embodying the metaphor of fluid thinking enhanced convergent thinking, which was measured by the ability to connect remotely associated concepts (Slepian & Ambady, 2012). Squeezing a ball with the left hand (Goldstein et al., 2010) or squeezing a hard ball with the dominant hand (Kim, 2015) also improved convergent thinking. Although walking was more beneficial for divergent thinking as compared to sitting, restrictions during walking negatively impacted divergent thinking (Murali & Händel, 2022). Last, whereas open expansive postures had a positive effect on divergent thinking, closed contractive postures were more advantageous for convergent thinking (Michinov & Michinov, 2022). These studies show that movement, alterations in body posture, and embodiment of concepts generally have a beneficial effect on creativity, but as in the case of Michinov and Michinov (2022), the benefits of the type of embodiment could be dependent on the type of creative thinking targeted. In this paper, we focus on a specific type of movement and embodied cognition—the case of *hand gestures*.

### Gestures for thinking and speaking

Many of us gesture spontaneously while we speak. These are called co-speech gestures and are different from the well-known “nonverbal behavior,” such as body posture or facial expressions. Co-speech gestures are closely synchronized and dependent on the content of speech, both in semantic and temporal terms (Bergmann et al., 2011; McNeill, 1992). Depending on the properties they serve, gestures can be classified into four different categories, as proposed by McNeill (1992): (1)* deictic/pointing* gestures—used to refer to a concrete or an abstract object; (2)* beat gestures*—rhythmic hand movements generally used to emphasize the discourse structure; (3)* iconic gestures*—used to convey the meaning of concrete objects, actions or events that are referred to in the speech (e.g., flapping one’s arms to represent a bird); (4)* Metaphoric gestures*—used to represent abstract rather than concrete concepts (e.g., an upward movement of the hand to represent a promotion at the workplace (Khatin-Zadeh et al., 2022)).

Co-speech gestures are not only but most frequently used when the listener is visible to the speaker (Alibali et al., 2001), when word retrieval is difficult (Krauss, 1998), when there is spatial imagery involved (Hostetter & Alibali, 2007), and overall, when there is a complex cognitive or linguistic task to be executed (e.g., Clough & Duff, 2020; Kita & Davies, 2009). Studies show that we use gestures to communicate with others but also to conceptualize our own ideas, even in the absence of a listener (Kita et al., 2017; Pine et al., 2010). One of the most prominent functions of gestures can be to facilitate the internal cognitive processes of the speaker, such as during word retrieval, by activating conceptual representations (Hadar & Butterworth, 1997; Krauss et al., 2000). The *Gesture-for-Conceptualisation Hypothesis* proposed by Kita et al. (2017) also suggests that gestures, and in particular representational gestures (i.e., iconic, metaphoric, and deictic gestures) support thinking and speaking by *activating*, *manipulating*, *packaging*, and *exploring* spatio-motoric information. Finally, gestures lighten the cognitive load (e.g., Goldin-Meadow, 2001) by externalizing the processing of information, distributing it to multiple modalities, and making the absent present (Ping & Goldin-Meadow, 2010). Therefore, gesturing might allow individuals to convey complex concepts and ideas more effectively. Moreover, the integration of gestures and speech could free up the cognitive load and help individuals think more elaborately and creatively.

### Gestures for creative thinking

Both the embodied cognition account (Barsalou, 1999; Sweller, 1988) and the gesture frameworks (e.g., Hostetter & Alibali, 2018; Kita et al., 2017) suggest that when people use gestures while speaking, they create and externalize the representations of the concepts they have in their minds. Moreover, they suggest that our cognitive processes are influenced by our bodily experiences and sensorimotor processes. Research has shown that bodily experiences such as gestures are associated with and can facilitate flexible and creative thinking (e.g., Frith, 2020; Kirk & Lewis, 2017; Laurent et al., 2020). This may happen in two ways. First, gestures aid retrieval ability, which can contribute to creative thinking, and second, gestures activate mental images that can facilitate creative imagination. Retrieval ability is important for creative thinking because generating novel and useful ideas or connecting remote associations primarily involves a strategic search through one’s semantic memory and exploring associational pathways (Beaty et al., 2014; Benedek et al., 2012; Silvia et al., 2013). Although the cognitive mechanisms underlying the association between imagery and creativity need further empirical investigation, the ability to generate vivid mental images is related to enhanced performance on creativity tasks and the real-life creative success of artists and inventors (May et al., 2020; Vallera & Gavard-Perret, 2012). Therefore, gestures are likely to boost creative thinking through the retrieval of concepts and words but also through activating vivid mental images.

Empirical studies with children found that children who were encouraged to gesture came up with more ideas on a divergent thinking task as compared to children who were not (Kirk & Lewis, 2017). However, these ideas were not necessarily more original or flexible, and restricting hand movements did not impair divergent thinking. In a different study, spontaneous gesture use was positively related to children’s narrative production (Laurent et al., 2020). Children who gestured told longer and more creative stories.

Studies with adults investigating the gesture-creativity relationship yielded some mixed findings depending on gesture types, measures of creativity, and individual differences in cognitive functioning, which is a quest for a further and more elaborate investigation. For example, baseline iconic gestures in adults were positively associated with four dimensions of divergent thinking -  *fluency*, *flexibility*, *originality (measured by rarity of responses)*, and *originality (measured by subjective rater scoring)* (Frith, 2020)*.* However, encouraging or restricting gesture use had an effect only on originality depending on one’s baseline gesture use. More specifically, individuals who were frequent gesturers (measured by their baseline condition) were less original (measured by subjective scoring) when their hands were restricted compared to when their gestures were encouraged, or when they executed meaningless hand movements. Interestingly, infrequent gesturers showed higher originality (measured by the rarity of responses) when their hands were restricted. Overall, these findings showed that encouraging or restricting gestures only affects originality in divergent thinking, and whether gestures benefit the originality of ideas or not depends on one’s natural inclination to gesture. A subsequent study measuring only originality in divergent thinking found that encouraging or restricting gestures did not influence originality (Fontenot, 2021). Responses were scored according to the Subjective Scoring Method (Silvia et al., 2013) and the extent of originality was based on *remoteness* (the distance from the intended use of a stimulus), *rareness* (how often that particular response occured within the sample) and *ingeniousness* (how ‘clever’ the response was). These limited and inconclusive findings require further research on the relationship between co-speech gestures and divergent thinking.

Only one study investigated the relationship between spontaneous and encouraged co-speech hand gestures and convergent thinking (Hyusein & Göksun, 2023). The results of that study showed that gesture production was associated with verbal, but not visual convergent thinking. Most importantly, both spontaneous and encouraged gestures negatively predicted convergent thinking for individuals with low mental imagery but had a positive effect for individuals with high mental imagery. Individual gesture analysis, namely representational vs. beat, showed that representational gestures were overall more beneficial for convergent thinking than beat gestures and these benefits would depend on individuals’ mental imagery skills. Apart from individual differences in gesture use, individual variations in mental imagery could also impact when and how someone benefits from gesturing for creative thinking. As evidence of such relationships has already been emerging for convergent thinking, there is a need to examine such effects of imagery and gestures also during divergent thinking problem-solving.

### Mental imagery in creative thinking and gesture production

An association between mental imagery and creativity has previously been established, e.g. nine hours of mental imagery training improved divergent flexible thinking and domain-specific creativity in dance students (May et al., 2020), individuals with more vivid imagery performed better on a creative drawing task (Vellera & Gavard-Perret, 2012) and instructions to produce mental images resulted in more fluent, flexible and original ideas (Vellera & Gavard-Perret, 2016). Furthermore, we have previously discussed a study indicating that the interaction between gestures and imagery predicts creative success in convergent thinking (Hyusein & Göksun, 2023).

The task used to measure mental imagery by Hyusein and Göksun (2023) —the Mental Imagery Test (MIT; Di Nuovo et al., 2014), entails the manipulation, such as mentally rotating a cube or the hands of a clock, and inspection of objects, such as estimating the distance between objects on a previously seen map. These skills are closely related to spatial imagery. We know from the literature that gestures, apart from being communicative tools, are also a mechanism for spatial representation and visualization (Hostetter & Alibali, 2007; Kita et al., 2017). For example, Eielts et al. (2020) found that gesturing benefited complex problem-solving for participants with lower visual working memory (a proxy for visual mental imagery), but not for participants with lower spatial working memory (a proxy for spatial mental imagery). Similarly, Hyusein and Göksun (2023) found that spatial imagery but not object imagery, as measured by a self-report questionnaire, had similar findings with imagery skills measured by the MIT. That is, encouraging gesture use in high spatial imagers benefitted convergent thinking, while encouraging gestures in low spatial imagers impaired convergent thinking. Therefore, the role of spatial skills, which are embedded in mental imagery, for the different domains of creativity should not be undermined and should be studied more extensively.

## The present study

Based on the findings that gestures and imagery skills of young adults interact in contributing to convergent creative thinking (see Hyusein & Göksun, 2023), the aim of the current study is to examine whether this is also the case for another type of creative problem-solving skill—divergent thinking. Frequency and types of spontaneous and encouraged iconic gestures will be examined in relation to solving a well-known divergent thinking task—Guilford’s Alternative Uses Test (AUT; Guilford, 1967). We will focus on iconic gestures as these are the types of gestures previous studies investigated or had promising findings about (e.g., Frith, 2020; Hyusein & Göksun, 2023; Kirk & Lewis, 2017). Moreover, iconic gestures are closely related to mental imagery (Hostetter & Alibali, 2018; Kita et al., 2017) and to the responses that the AUT is expected to elicit. Additionally, to determine the role of mental imagery skills in the gesture-divergent thinking relationship, we will use the Mental Imagery Test (MIT; Di Nuovo et al., 2014), which assesses the maintenance, inspection, generation, and manipulation of different categories of images.

As there are large individual differences in gesture rate use across individuals (Hostetter & Alibali, 2007; Özer & Göksun, 2020), this study will employ both a between- and a within-subjects design. One group of participants will be exposed to the AUT without any mention of hand movements, and later, solve the AUT with different objects while encouraged to gesture. A second group of participants will solve the AUT only while being encouraged to gesture. The within-subject effects will allow for larger power in detecting the effect of condition by accounting for the individual variations in gesturing, and the between-subject condition will help us disentangle practice effects from the effects of encouraging gestures.

There are two main hypotheses in the current study:

1. First, spontaneous iconic gestures will predict all of the components of divergent thinking in the gesture-spontaneous condition. This is based on Frith’s (2020) study where baseline gesture frequency was associated with fluency, flexibility, and originality of ideas. Elaboration was not measured in their study. However, Laurent et al. (2020) found that preschoolers who have higher spontaneous representational gesture frequency tell longer and more creative narratives. As elaboration is related to verbally expressing more details related to creative ideas, we also expect spontaneous gestures to predict elaboration.
  Moreover, we hypothesize that encouraged iconic gestures will predict fluency and elaboration but not originality or flexibility of ideas. Fluency and elaboration in contrast to originality and flexibility are measures that are more sensitive to one’s general vocabulary or verbosity (Zhang et al., 2020). As theoretical perspectives, such as the Growth-Point-Theory (McNeill, 2013), propose that speech and gesture are tightly integrated processes originating from the same system, we expect an increased gesture rate to be more likely to lead to increased verbal fluency and elaboration. This hypothesis is also supported by previous findings. For example, people who were encouraged to gesture told longer narratives (Cravotta et al., 2019). In addition, encouraging iconic gestures increased fluency in children’s creativity, however, it did not boost flexibility or originality (Kirk & Lewis, 2017). This finding was further replicated in younger adults by Frith (2020). A reason why originality and flexibility might not be enhanced by encouraged gesture use is because they might be more dependent on factors unrelated to speech. For example, originality is mostly related to experiential bias (Runco & Acar, 2010) and inhibition, while flexibility has been associated with switching and shifting abilities (for a review, see Palmiero et al., 2022).
  The difference expected in spontaneous vs. encouraged iconic gesture rate for flexibility and originality is based on the fact that when people gesture spontaneously, they might be relying on their natural inclination for using their verbal and visuo-spatial resources, which gestures are a part of. Thus, the more they gesture, the more advantage they take of their resources, which leads to increased flexibility and originality. However, further gesture encouragement would not lead to higher flexibility or originality because these two specific components of divergent thinking are more reliant on different cognitive and experiential factors as described above (Palmiero et al., 2022; Runco & Acar, 2010).
2. We expect an interaction among gestures, mental imagery, and divergent thinking. Similar to convergent thinking (Hyusein & Göksun, 2023), we hypothesize that gestures will enhance divergent thinking for high-imagery individuals but will not have the same effect for low-imagery individuals.

## Methods

### Participants

A total of 90 young adults (*M*_age_ = 21.4, SD = 2.46; 57 females) took part in the current study. All of them were Turkish native speakers. Forty-eight of the participants were undergraduate students who were recruited through Koç University’s subject pool for partial fulfilment of course credit. The rest of the young adults were recruited based on convenience sampling. The data of nine participants were excluded due to technical errors and one participant was discarded because they reported having an advanced visual disability during the testing procedure. Therefore, the data of 80 participants were included in the final analysis (*M*_age_ = 21.3, SD = 2.46; 52 females). On recruitment, participants were randomly assigned either to Group 1 (*N* = 40) where they completed both gesture-spontaneous and gesture-encouraged conditions or to Group 2 (*N* = 40) where they only completed the gesture-encouraged condition. The sample size was determined based on previous studies that investigated the effects of gesture use on creative thinking (Kirk & Lewis, 2017; Liao & Wang, 2019). In Kirk and Lewis (2017), the sample size was calculated based on power of 0.80, which was sufficient to detect an effect with a magnitude (*d*_*z*_) of 0.5. Ethical approval was granted by Koç University Ethical Committee on Human Research (IRB Protocol No. 2020.125.IRB3.063).

### Materials

#### Divergent thinking

##### Alternative uses test (AUT; based on Guilford, 1967)

This is a test of divergent thinking where participants were asked to come up with as many possible uses of an everyday object as they could in one minute. We used four objects (newspaper, pencil, shoe, towel) in total—two for each condition, in a counterbalanced order. Scoring was based on four components:

##### Fluency

The total number of ideas/responses given by the participant. Each response received 1 point.

##### Originality

Each response was compared to the total amount of responses from all of the subjects. Responses that were given by only 5% of the group were counted as unusual (1 point) and responses given by only 1% of them were counted as unique (2 points).

##### Flexibility

The number of different categories the participant’s responses belonged to. Each unique category received 1 point. If a response belonged to a category that was previously assigned to another response for that specific object, it received a score of 0.

##### Elaboration

The amount of detail, e.g., “a boat” counts 0, whereas “as a boat, by putting toys in it, in a pool” counts 2 (1 point for an explanation of ‘putting toys in it’ and another for further detail about ‘in a pool’).

#### Mental imagery

##### Mental imagery test (MIT; Di Nuovo et al., 2014)

This is a battery of eight tasks designed to measure mental imagery skills, namely the *generation*, *maintenance,* and *manipulation* of different categories of images. For example, in one of the tasks participants are asked to imagine a clock with hands indicating 10 past 10, then imagine the clock reflected in a mirror and tell the time reflected in the mirror (answer—1:50 or 10 to 2). Then, they are asked to say the time reflected in the mirror after 10 min (answer—1:40 or 20 to 2). In another task, they are shown a large cube composed of nine small cubes per face (3 × 3) where the external faces are colored. After looking at the cube for 30 s, participants are asked to say the number of small cubes that have three/two/one/none colored faces without looking at the cube. This task was used before in Turkish samples with Turkish instructions (Arslan & Göksun, 2020; Hyusein & Göksun, 2023). The task stimuli were presented on a white screen and answers were noted down on the answer sheet by the experimenter during the sessions. A total score of mental imagery was calculated by adding up the scores of the eight tasks. On the MIT, a participant could achieve the maximum total score of 83.

This study was part of a larger project related to creativity and gestures. Experiments were conducted online in one-to-one sessions on Zoom. The data were collected by the first author and three trained research assistants. All sessions were video recorded for further speech transcription and gesture coding. After obtaining verbal informed consent, the participant was instructed to adjust their body position so that their upper body, arms, and hands could be captured by the camera.

Participants in Group 1 completed the AUT twice—first in a gesture-spontaneous condition (no mention of gestures or hand movements), and after that, in a gesture-encouraged condition where we explicitly encouraged them to use their hands while thinking and speaking. Participants in Group 2 completed the AUT only in the gesture-encouraged condition. Testing Group 2 enabled us to have both a within-subject and a between-subject design thus disentangling learning or practice effects from the effects of gesturing alone. We used identical stimuli for Group 1 and Group 2 in a counterbalanced order across groups and conditions. The instructions of the gesture-encouraged condition were as follows: “Please, use your hands while speaking and thinking when solving the next task.” Instructions were given before the task. No particular type of gesture was given as an example.

Following the AUT, participants completed the MIT. After that, the recording was paused, and participants completed a demographic form on Qualtrics. Finally, participants were debriefed and thanked for their time or awarded course credits if they were recruited through the university’s subject pool system. Depending on the group the participant was assigned to, and as this experiment was part of a larger project involving other cognitive measures, the duration of the sessions varied between 40 and 60 min.

#### Coding and scoring

##### AUT

AUT responses were transcribed on Microsoft Excel by the first author and a trained research assistant. Each idea was transcribed under a “uses” column and the details were transcribed under a separate “details” column. The number of uses represented the fluency score for each item and the number of details represented the elaboration score for each item. The responses were assigned into relevant categories and the number of different categories used for each item made up the flexibility score for that item. When all responses were transcribed, the originality scores were calculated as described in the Materials section.

Interrater reliability was calculated for fluency, flexibility, and elaboration for 20% of the data, which was coded independently by the first author and a trained research assistant. Inter-rater intra-class correlation coefficients (ICC) indicated excellent reliability for all three measures, ICC_fluency_ = 0.971, 95% CI [0.936, 0.989], *p* < 0.001; ICC_flexibility_ = 0.970, 95% CI [0.934, 0.985], *p* < 0.001; ICC_elaboration_ = 0.968, 95% CI [0.942, 0.984], *p* < 0.001. As the originality score was calculated according to a standardized procedure, there was no need to assess interrater reliability for it.

#### Gesture

Speech and gestures for the AUT were transcribed and coded on Microsoft Excel by a trained research assistant. Another trained research assistant checked the gesture coding, and inconsistencies were resolved by the first author. In addition to the McNeill-type co-speech gestures, i.e., iconic, deictic, metaphoric, and beat, palm-revealing gestures were also coded (Chu et al., 2014; McNeill, 1992). We coded palm-revealing gestures because an earlier study of gestures and creativity (i.e., Hyusein & Göksun, 2023) has included them in their coding system. We also coded “other” gestures, which included emblems and gestures that did not belong to the categories mentioned above. All gesture types were mutually exclusive from each other. A third independent assistant coded 20% of the data for interrater agreement. There was excellent reliability between the two coders (ICC = 0.910, 95% CI [0.869, 0.939], *p* < 0.001). Gesture frequency was calculated for each gesture type by dividing the number of gestures produced by the number of words spoken in each trial. We used gesture frequency rate and not the sheer number of gestures produced because as people talk more, the number of gestures also increases; therefore, calculating a gesture rate is beneficial for controlling individual differences in speech production (So et al., 2009). For the sake of parsimony and alignment with the goal of the current study, only *iconic gesture* frequency was included in the analyses.

The data are available at the Open Science Framework: https://osf.io/c7umf/?view_only=3e2eef623843477f8ccb86dcdcde02cb

## Results

The mean and standard deviation values for age, the divergent thinking measures, namely *fluency*, *originality*, *flexibility*, and *elaboration,* and iconic gesture frequency are presented in Table 1. Descriptive statistics for the other gesture types (i.e., beat, iconic, metaphoric, deictic, palm-revealing gestures and total gesture frequencies) can be found in the Supplementary Materials (Table S1). Spearman correlation matrices for each condition and group (i.e., Group 1 gesture-spontaneous condition, Group 1 gesture-encouraged condition, and Group 2 gesture-encouraged condition) can also be found in the Supplementary Materials (Tables S2–S4).

**Table 1:** Mean (*M*) and standard deviation (*SD*) values of age, AUT/divergent thinking measures, and iconic gesture frequency across groups and conditions

	Groups					
	Gesture-spontaneous Group 1 (GS1)		Gesture-encouraged Group 1 (GE1)		Gesture-encouraged Group 2 (GE2)	
	*M*	*SD*	*M*	*SD*	*M*	*SD*
AUT fluency	6.09	2.12	5.90	1.81	5.63	2.29
AUT originality	2.48	1.97	2.49	1.94	2.44	2.33
AUT flexibility	4.31	1.56	4.06	1.24	3.63	1.53
AUT elaboration	5.94	3.14	7.09	3.77	5.26	3.13
Iconic gesture frequency	0.08	0.07	0.13	0.09	0.09	0.08

To test our hypotheses regarding the effect of iconic gesture use and mental imagery skills on divergent thinking, we used multi-level linear mixed effects modeling (lmer). We included random intercepts for Subject and Item for our between-group analyses, i.e., gesture-encouraged condition group 1 (GE1) and gesture-encouraged condition group 2 (GE2), and a random intercept for Item and a random slope of Condition by Subject for our within-group analyses, i.e., gesture-spontaneous condition group 1 (GS1) and gesture-encouraged condition group 1 (GE1). The mixed-effects approach allowed us to account for the random variability of different participants and different items while with the random slope of Condition by Subject we tested for within-subject effects. We built separate models for each divergent thinking measure (*fluency*, *originality*, *flexibility,* and *elaboration*). Our outcome variables were the divergent thinking measures while iconic gesture frequencies and mental imagery scores were included as predictors. Each predictor was added incrementally as a fixed effect to the model and compared to the previous model to test whether the inclusion of the new predictor significantly improved the model or explained a significantly greater proportion of the variance. We always started with a null model (for the between-subject effects—a random item- and participant-intercept-only model, and for the within-subject effects—a random slope of condition by participant and a random item-intercept-only model), then added one fixed effect and compared the new model to the null model. Each predictor was added incrementally to the model and the new model was compared to the previous one which contained all the previous predictors except the one added at this step. For the within-subject analyses, after building the null model, we added condition as a fixed effect, then iconic gesture frequency to the previous model, and finally mental imagery scores. For the between-subject analyses, instead of condition we added group. After each main-effects model, two-way and three-way interaction models were also tested and compared to the main-effects models. We used chi-square tests (likelihood ratio tests) to test model significance during model comparison and the *summary*() function to obtain regression coefficients and *p*-values for the predictor variables. To understand interaction effects for continuous variables we used the *sim_slopes* function from the *jtools* package and the *emmeans* function from the *emmeans* package for categorical variables. We tested the normality assumption of each incremental model with the Shapiro–Wilk test. When normality was violated, we bootstrapped each model with the *bootstrap*() function of the *lmeresampler* package. The number of bootstrap iterations was set to 1000. All continuous variables were normalized with the *scale*() function prior to building the linear mixed models. Normalizing or standardizing continuous data would allow us to compare variables that are not measured in the same way, equate their variance, and reduce multicollinearity among the independent variables. Analyses were performed with the lme4 and lmerTest packages (Bates et al., 2015) in R (R Core Team, 2013).

### Fluency

#### Within-group analyses (GS1 and GE1)

None of the fixed effects, namely condition, iconic gesture frequency, or mental imagery that were added incrementally to the null model were significant. Two- and three-way interactions were also not significant. The normality assumption of each model was not violated.

#### Between-group analyses (GE1 and GE2)

Adding the main effect of iconic gestures to the group-only model significantly improved the model, *χ*^2^(1) = 9.23, *p* = 0.002. Iconic gesture use was a positive predictor of fluency for both Groups 1 and 2 (see Table 2). That is, the more iconic gestures people produced when encouraged to gesture, the more fluent they were irrespective of their group [irrespective of whether they had practice (Group 1) with the AUT task or not (Group 2)]. Adding mental imagery, a two-way interaction between group and iconic gestures or a three-way interaction among group, iconic gestures, and mental imagery did not improve the main effects model of group and iconic gestures. Normality assumptions were not violated.

**Table 2:** Model summary for between-group (GE1 and GE2) analyses of fluency

	Coefficient	SE
Fıxed effects		
Intercept	− 0.07	0.20
Group	0.03	0.22
Iconic gesture frequency	0.24**	0.08

	Variance	SD
Random effects		
Intercepts		
Subject	0.51	0.71
Item	0.04	0.20

Significance codes = ***p < 0.001, **p < 0.01, *p < 0.05, ^†^*p* < 0.1

### Originality

#### Within-group analyses (GS1 and GE1)

The null model of originality violated the normality assumption, *W* = 0.95, *p* < 0.001, therefore, bootstrapping was applied. As the bootstrap test compares the fitted model to reduced models, we then built a final model including the three-way interaction of condition, iconic gestures, and mental imagery, and applied bootstrapping to it. The interaction between condition and iconic gestures was significant, 95% CI [− 0.75, + 0.02]. However, when we conducted simple slope analysis, none of the slopes were significant, *β* = − 0.22, SE = 0.15, *p* = 0.13 for GS1 and *β* = 0.16, SE = 0.11, *p* = 0.15 for GE1. Even though there was a trend for spontaneous iconic gestures to be negative predictors of originality while iconic gestures in the gesture-encouraged condition to be positive predictors, these effects did not reach significance.

#### Between-group analyses (GE1 and GE2)

Similar to the within-group analysis, the normality assumption of the models for originality was violated, *W* = 0.96, *p* < 0.001 (Shapiro–Wilk test of the null model) and bootstrapping was applied. No significant effects were detected in the three-way interaction model of group, iconic gestures, and mental imagery. However, in the model with the main-effects-only, iconic gestures were a significant predictor of originality, 95% CI [0.01, 0.37] (see Table 3). This implies that when encouraged, iconic gestures were positively related to originality irrespective of imagery skills or experience with the task.

**Table 3:** Model summary for between-group (GE1 and GE2) analyses of originality

	*b*	*b* 95% CI [LL, UL]
Fıxed effects		
Intercept	0.03	[− 0.27, 0.33]
Group	0.01	[− 0.41, 0.44]
Iconic gesture frequency	0.19*	[0.01, 0.37]
Mental ımagery	− 0.03	[− 0.24, 0.17]

	Variance	*SD*
Random effects		
Intercepts		
Subject	0.30	0.54
Item	0.00	0.00

Significance codes = ***p < 0.001, **p < 0.01, *p < 0.05, ^†^*p* < 0.1

### Flexibility

#### Within-group analyses (GS1 and GE1)

**Fig. 1:**
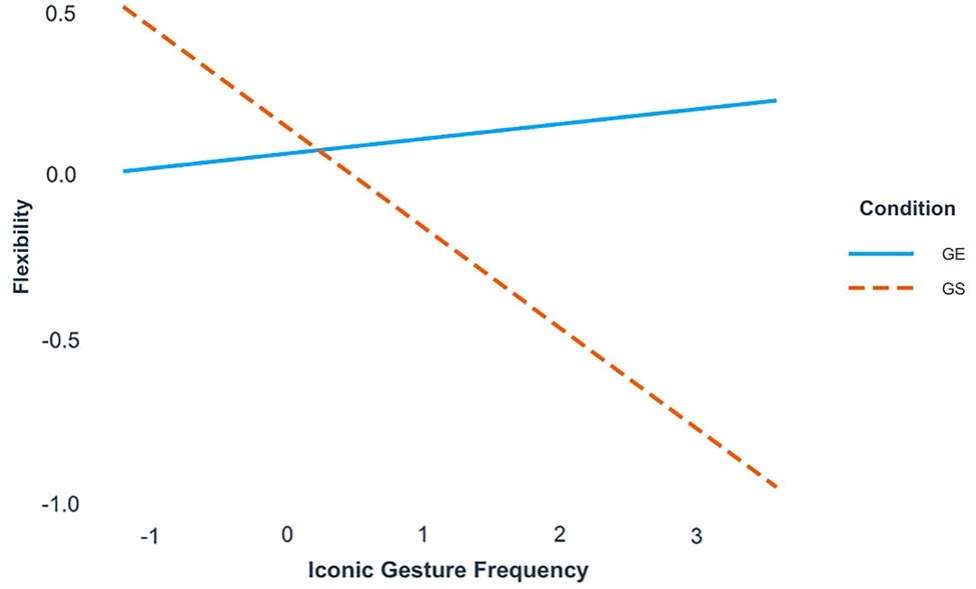
Interaction plots for scaled flexibility scores and scaled iconic gesture frequency values in Group 1 (gesture-encouraged (GE) and gesture-spontaneous (GS) conditions) (colour figure online)

The interaction between condition and total gesture frequency of Group 1 significantly improved the initial main effects model, *χ*^2^(1) = 4.66, *p* = 0.03. The normality assumption of the model was not violated, *W* = 0.98, *p* = 0.13. We found a significant interaction between condition and iconic gestures (see Table 4). Simple slope analysis revealed that the slope of iconic gesture frequency for the gesture-spontaneous condition of Group 1 was significant, *β* = − 0.30, SE = 0.13, *p* = 0.02, which means that the more iconic gestures people produced spontaneously, the less flexible their ideas were (see Fig. 1). The slope of the gesture-encouraged condition was not significant, *β* = 0.06, SE = 0.08, *p* = 0.60, meaning that encouraging gesture use did not affect flexibility.

**Table 4:** Model summary for within-group (GS1 and GE1) analyses of flexibility

	Coefficient	*SE*
Fıxed effects		
Intercept	0.06	0.22
Condition	0.08	0.29
Iconic gesture frequency	0.04	0.08
Condition*ıconic gesture frequency	− 0.35*	0.15

	Variance	*SD*
Random effects		
Intercepts		
Subject	0.16	0.40
Item	0.06	0.25
Slopes		
Condition|subject	0.14	0.37

Significance codes = ***p < 0.001, **p < 0.01, *p < 0.05, ^†^*p* < 0.1

Adding the main effect of mental imagery did not improve the two-way interaction model, *χ*^2^(1) = 0.00, *p* = 0.97, neither did a model with a three-way interaction, *χ*^2^(3) = 3.01, *p* = 0.39. However, the normality assumption of the model including the three-way interaction was violated, *W* = 0.98, *p* = 0.04. After bootstrapping the final model, we found a significant two-way interaction between condition and iconic gestures, 95% CI [− 0.75, + 0.08], which was in line with the two-way interaction model reported in Table 4.

#### Between-group analyses (GE1 and GE2)

No significant effects or models were detected for the between-subject analysis of originality. None of the models violated the normality assumption.

#### Elaboration

##### Within-group analyses (GS1 and GE1)

The main effect model of iconic gestures significantly improved the condition-only model, *χ*^2^(1) = 7.90, *p* = 0.005. There was a main effect of iconic gestures, *β* = 0.19, SE = 0.07, *p* = 0.005, meaning that the more iconic gestures people used irrespective of condition, the more elaborate their ideas were (see Table 5). The normality assumption of the model was not violated, *W* = 0.99, *p* = 0.30. Neither a two-way interaction between condition and iconic gestures nor adding mental imagery skills as a main effect or as an interaction term improved the model. The within-subject results for elaboration and iconic gestures showed that producing iconic gestures was related to increased elaboration of ideas independent of the gesture condition.

**Table 5:** Model summary for within- and between-group analyses of Elaboration

	Within-group (Group 1)		Between-group (Group 1 and Group 2)	
	Coefficient	*SE*	Coefficient	*SE*
Fıxed effects				
Intercept	0.26	0.31	0.26	0.21
Condition	− 0.21	0.38		
Group			− 0.44	0.24
Iconic gesture frequency	0.19**	0.07	0.18^*^	0.08

	Variance	*SD*	Variance	*SD*
Random effects				
Intercepts				
Subject	0.85	0.92	0.55	0.74
Item	0.13	0.36	0.05	0.22
Slopes				
Condition|subject	0.15	0.39		

Significance codes = ***p < 0.001, **p < 0.01, *p < 0.05, ^†^*p* < 0.1

##### Between-group analyses (GE1 and GE2)

A main-effects model of iconic gesture frequency significantly improved the group-only model, *χ*^2^(1) = 4.71, *p* = 0.03. Only the main effect of iconic gestures was significant, *β* = 0.18, SE = 0.08, *p* = 0.03, which revealed that iconic gesture frequency positively predicted elaboration of ideas in both gesture-encouraged conditions (see Table 5). The normality assumption was not violated, *W* = 0.98, *p* = 0.08. Adding the main effect of imagery or the interaction terms did not improve the model.

Therefore, iconic gestures were positive predictors of elaboration of ideas irrespective of whether they were encouraged or not or whether participants had previous exposure to the AUT task or not.

## Discussion

The purpose of the current study was to gain a better understanding of how iconic gesture use was related to the subcomponents of divergent thinking and test whether mental imagery skills contributed to the gesture—divergent thinking relationship. We found that: (1) *fluency, originality,* and *elaboration* were positively associated with iconic gestures when gesture use was encouraged; (2) while *flexibility* was negatively predicted by spontaneous iconic gestures, *elaboration* was positively predicted by them; (3) *mental imagery skills* did not play a role in the gesture—divergent thinking association.

### Encouraged iconic gestures were positively related to creative fluency and originality

Our first hypothesis that encouraging gesture use would lead to enhanced fluency and elaboration and spontaneous iconic gestures would be positively associated with originality and flexibility in divergent thinking was partially supported.

First, iconic gestures predicted fluency of ideas when gestures were encouraged but not when they were spontaneous. Moreover, encouraging gestures did not lead to higher fluency in the group that attended both the gesture-spontaneous and the gesture-encouraged conditions. This could mean that when baseline iconic gesture frequency and/or baseline AUT fluency scores were taken into account (by conducting the within-group analysis), encouraged gestures did not in fact improve fluency. This finding is in line with Frith (2020) who also did not find that hand movement manipulation (encouraging or restricting) led to changes in fluency. However, when we tested the effects of encouraged gestures (between-group analysis) on fluency, we found that iconic gestures were positive predictors of fluency. Therefore, we can conclude that encouraging gestures might have positive effects on fluency of ideas, but this finding should be taken with caution. In other words, iconic gestures have the potential to boost fluency, but it might be that they only help depending on people’s baseline gesture rate.

Next, similar to fluency, iconic gestures were also a positive predictor of originality when gestures were encouraged in the between-subject analysis. Put differently, when people produced iconic gestures in their encouraged conditions, the more iconic gestures they produced, the more original their ideas were. For the group that also participated in the spontaneous gesture condition, we found a significant interaction between iconic gestures and condition, such that when people gestured spontaneously iconic gestures were negatively associated with originality but when people were encouraged to gesture iconic gestures were positively associated with originality. However, the slopes of these effects were not significant. Therefore, it is more sensible to conclude that as in the case of fluency, when individual differences in baseline iconic gesture use are taken into consideration, encouraging gestures might not benefit originality in the same way for everyone.

At the same time, detecting a significant interaction for spontaneous and encouraged gestures, even if the slopes were not significant, shows that the beneficial effect of iconic gestures might be stronger for originality compared to fluency. So far, the only study that has found an association between gesture and originality was the doctoral thesis by Frith (2020). In her study, Frith (2020) classified participants according to their baseline gesture rates and found that gesture restriction could benefit or hamper originality depending on whether one is a frequent or infrequent gesturer. Even though we also used a within-subjects design taking into account individuals’ spontaneous gesture use, we did not use a gesture-restricted condition. Notice that Kirk and Lewis (2017) who used a gesture-restricted condition for children’s idea generation did not find an effect of the restricted condition on divergent thinking.

One reason for these inconsistent findings could also be the way originality is measured. In the aforementioned studies, including the present one, it varied from subjective rater-based assessment to purportedly more objective “statistical uncommonness of responses.” However, even the more objective statistical calculations of originality vary among each other (e.g., in some studies, two points are assigned for responses given by ≤ 10% of the sample, while in others by ≤ 1% of the sample). In our study, even though we used the latter more conservative scoring method, we still found an effect of encouraged gestures on originality, which highlights the promising potential of hand gestures for coming up with original ideas.

### Both spontaneous and encouraged iconic gestures were related to enhanced elaboration

We also demonstrated that iconic gestures were positively related to creative elaboration (i.e., the number of details people added to their ideas), and this was true both when they gestured spontaneously and when their gestures were encouraged. This is a strong finding showing that irrespective of how much people gesture spontaneously, encouraging gesture use always benefits creative elaboration. This finding could also be partially explained by previous research showing that instructing participants to gesture leads to an increase in discourse length, or longer narratives (Cravotta et al., 2019). Therefore, in our task producing more gestures might have led to more detailed and elaborate ideas. As in our study participants’ spontaneous gesture rate also predicted higher elaboration, it could also be the case that gesturers also produce more elaborate ideas in general. A similar finding was reported in a recent study with children—preschoolers who used representational gestures spontaneously told longer stories and used more creative (lower frequency words) compared to preschoolers who did not gesture (Laurent et al., 2020).

### Encouraging gestures mitigated the negative effects of spontaneous iconic gestures on flexibility

In contrast to our expectations, flexibility in divergent thinking was not positively associated with spontaneous iconic gestures. Here, we found some interesting patterns regarding flexibility and hand gestures. When people gestured spontaneously, gestures were a negative predictor of flexibility of ideas. The more iconic gestures people used when describing their ideas, the less flexible their ideas were. That is, their creative products belonged to less diverse categories. Even though less studied than the other dimensions of divergent thinking, flexibility is an important aspect that ensures capturing participants who generate truly diverging ideas (Acar & Runco, 2019; Grajzel et al., 2023). An interpretation of why iconic gestures were negative predictors of flexibility could be that iconic gestures might keep participants stuck in the same category of ideas by overemphasizing the features of the objects that are being described. Therefore, by using iconic gestures participants might come up with more elaborate ideas but these ideas or products usually belong to the same category and are less flexible. Interestingly, this effect is not present in the gesture-encouraged conditions. Therefore, an increase in iconic gesture frequency in the encouraged conditions (as was the case here) might have ameliorated the negative effect of iconic gestures for flexible idea generation. The mechanisms behind why spontaneous iconic gestures are negatively associated with flexibility and why encouraging gesture use mitigates those effects still need to be clarified. One possible reason could be that when people gesture spontaneously, they are more likely to gesture about one aspect of the object they are prompted with. For example, studies show that when participants are shown a picture rather than the name or a verbal description of the object, they have more trouble finding alternative uncommon uses of the prompt (Chrysikou et al., 2016) and are more likely to experience design fixedness/design fixation, i.e., they are not able to find alternative design solutions (Chrysikou & Weisberg, 2005). As gestures in a similar way might act as image activators, they might cause people to fixate on one category of uses. However, when encouraged to gesture, people might start to gesture more intentionally and in a more versatile manner activating different aspects of the prompted object. More qualitative gesture analysis, such as the aspects of the object that iconic gestures depicted, could help us decipher the meaning of our findings.

### Mental imagery did not moderate the relationship between gestures and divergent thinking

Our second hypothesis that gestures would be particularly beneficial for people with high imagery skills was not supported. We did not find a differential effect of imagery skills on any of the divergent thinking components. Whereas Hyusein and Göksun (2023) showed that imagery skills matter for how much people benefit from representational gestures in verbal convergent thinking tasks, that was not the case for divergent thinking. In the current study, individuals benefitted from gestures, irrespective of imagery skills. This finding might highlight the disparity between *divergent* and *convergent* thinking. Even though both of them have been frequently used synonymously to *creativity*, we should be careful when making implications. But why might it be the case that only high imagers benefit from gestures when solving convergent thinking problems and for divergent thinking, imagery skills are not of particular importance? Convergent thinking, which is the ability to connect remote associations (i.e., finding a common word to unrelated three words, e.g., *Swiss-cake-cottage* with the common word being *cheese*) might require a more strategic and controlled search in semantic memory compared to divergent thinking. The task used to measure mental imagery by Hyusein and Göksun (2023) also requires strategic and analytical thinking (e.g., mental rotations, geometrical computations, map search retrieved from memory, etc.). Therefore, imagery skills were not only highly predictive of success in convergent thinking but as gestures are reliant on imagery (see Hostetter & Alibali, 2007, Kita et al., 2017), an effective combination of imagery and gestures was needed to achieve good convergent thinking performance. On the contrary, divergent thinking is a more “scattered” cognitive process (Maheshvari et al., 2021). As in the AUT participants are not looking for the “right” answer, they need a less strategic search, which might be why imagery skills did not affect divergent thinking. The role of semantic knowledge in creative thinking has long been emphasized (Beaty et al., 2014). Since creative ideas come from searching, reorganizing, and combining semantic knowledge (Ovando-Tellez et al., 2022) and gestures activate implicit knowledge (Goldin-Meadow, 2001), semantics could be a potential mechanism influencing the interplay between gestures and divergent thinking. Therefore, along with imagery, semantics might be key to activating creativity when people use gestures. Such a link still needs to be tested.

One limitation of the study is that it lacked a gesture-restricted condition. Kirk and Lewis (2017) showed that restricting children from gesturing did not hinder their creative idea generation. However, Frith (2020) found that restricting hand movement could impact originality in different ways—it could enhance originality in infrequent gesturers but hinder it in frequent gesturers. A future direction would be to test if restricting gestures could also impact low- and high-imagers differently.

To sum up, we found that gestures have a promising role in enhancing divergent thinking irrespective of individuals’ mental imagery skills. We found the strongest effect for creative elaboration—both spontaneous and encouraged iconic gesture use predicted elaboration. Producing iconic gestures, when gesture use was encouraged, was also associated with greater fluency and originality of ideas. At the same time, we should take the findings regarding fluency and originality with caution as the effect of encouraged gestures was not present when spontaneous gestures were taken into account. Finally, even though flexibility was negatively associated with spontaneous iconic gestures, this negative association disappeared when gestures were encouraged, which again points to the beneficial role of encouraging gesture use for thinking and speaking.

### Theoretical implications

Our findings carry valuable implications for the embodied creativity account and the gesture for thinking and speaking frameworks (e.g., Hostetter & Alibali, 2018; Kita et al., 2017; Stanciu, 2015). By demonstrating that certain types of hand movements are related to and can influence the features of the ideas we generate, we provided empirical support that creative thinking can be embodied. Or, the way dance scholar Sheets-Johnstone (2013) has put it—there is “thinking in movement.” We support this claim by showing that iconic gestures are related to both enhanced but also reduced features of divergent thinking. Unlike convergent thinking, where the effect of gestures depended on one’s mental imagery (Hyusein & Göksun, 2023), here we did not find such an interaction, which leaves room for further exploration regarding the factors that play a role in the “dance” between gestures and divergent thinking.

Another account that is worth further exploration and is relevant to both embodied cognition theories and gesture hypotheses is investigating the way creativity could be affected by seeing other’s gestures and then, how being exposed to other’s gestures would affect one’s own gesture production during creative thinking. This is relevant to embodied cognition theories because others’ or the listeners’ gestures are also part of our physical environment. Additionally, there is plenty of research showing that gesture comprehension is an integral part of learning and cognition (for a review see Özer & Göksun, 2020). Therefore, to get a more comprehensive understanding of how gestures as embodied actions affect our creative cognition, we might need to study creative thinking in more interactive environments, such as group brainstorming sessions.

Finally, even though Frith (2020) investigated how meaningful (gestures) versus meaningless (moving hands in circles) movements affect divergent thinking, they did not provide a clear conclusion. Moreover, moving hands in circles resembles the fluid movement performed in previous studies (e.g., Imaizumi et al., 2020; Slepian & Ambady, 2012), which associated fluid movement with fluid creative thinking. Therefore, it is questionable how “meaningless” moving hands in circles could be. Future studies could propose more robust ways to measure meaningless movements in contrast to meaningful movements such as gestures or fluid hand movements. Such a line of research could elucidate the importance of different types of actions for creative embodied cognition.

## Conclusion

The current study provided evidence of the intricate nature of hand gesture use for thinking and cognition. By focusing on the actively debated topic of creative thinking and embodied cognition, we showed that hand gestures can spark more fluent, original, and elaborate creative ideas. Moreover, when used intentionally, they can boost the flexible divergent flow. By studying the process of how divergent creative ideas are linked to hand gestures, the present study also contributed to the call in creativity research, emphasizing the need to investigate creativity as a process rather than solely an end product. Along with providing empirical support for the multifaceted nature of gestures for divergent creative thinking, the current study also raised a variety of intriguing questions for future research. For example, why do unintentional (spontaneous) iconic gestures lead to functional fixedness, but intentional (encouraged) iconic gestures do not? If the mechanism behind gestures and convergent thinking relies on mental imagery but the one behind gestures and divergent thinking does not, what factors might play a role in divergent thinking? Such a line of research can have tremendous implications not only for psycholinguistic theories of multimodal communication, but also for the field of education where both teachers and students can make use of their gestures to foster creative thinking while teaching and learning.

### Supplementary Information

Below is the link to the electronic supplementary material.

Supplementary file 1 (DOCX 50 kb)

## Data Availability

The dataset is available at the Open Science Framework: https://osf.io/c7umf/?view_only=3e2eef623843477f8ccb86dcdcde02cb.
