# Supplementary material for: Give your ideas a hand: the role of iconic hand gestures in enhancing divergent creative thinking
Source: Psychol Res. 2024 Mar 28;88(4):1298–313. doi: 10.1007/s00426-024-01932-1 (PMC11142943; doi:10.1007/s00426-024-01932-1)
Supplement: Supplementary file 1 — (DOCX 50 kb) [file 426_2024_1932_MOESM1_ESM.docx]

**Table S1.** Mean (*M*) and standard deviation (*SD*) values of beat, metaphoric, deictic and palm-revealing gesture frequencies across groups and conditions.

|  | Groups | | | | | |
| --- | --- | --- | --- | --- | --- | --- |
|  | Gesture-Spontaneous Group 1 (GS1) | | Gesture-Encouraged Group 1 (GE1) | | Gesture-Encouraged Group 2 (GE2) | |
|  | ***M*** | *SD* | ***M*** | *SD* | ***M*** | *SD* |
| Beat Gesture Frequency | **0.04** | 0.05 | **0.05** | 0.05 | **0.05** | 0.06 |
| Metaphoric Gesture Frequency | **0.01** | 0.02 | **0.02** | 0.02 | **0.02** | 0.03 |
| Deictic Gesture Frequency | **0.00** | 0.01 | **0.01** | 0.02 | **0.01** | 0.03 |
| Palm-revealing Gesture Frequency | **0.01** | 0.02 | **0.02** | 0.03 | **0.02** | 0.05 |

|  | | | | | | | | | | | | | | | | | | | | | | | | | | | | |
| --- | --- | --- | --- | --- | --- | --- | --- | --- | --- | --- | --- | --- | --- | --- | --- | --- | --- | --- | --- | --- | --- | --- | --- | --- | --- | --- | --- | --- |
|  | |  | | **AUT** | | **Fluency** | | **Originality** | | **Flexibility** | | **Elaboration** | | **Total Gestures** | | **Beat** | | **Iconic** | | **Metaphoric** | | **Deictic** | | **Palm-revealing** | | **MIT** | |  |
| **AUT** |  | Spearman's rho |  | — |  |  |  |  |  |  |  |  |  |  |  |  |  |  |  |  |  |  |  |  |  |  |  |  |
|  |  | p-value |  | — |  |  |  |  |  |  |  |  |  |  |  |  |  |  |  |  |  |  |  |  |  |  |  |  |
| **Fluency** |  | Spearman's rho |  | 0.864 | *** | — |  |  |  |  |  |  |  |  |  |  |  |  |  |  |  |  |  |  |  |  |  |  |
|  |  | p-value |  | < .001 |  | — |  |  |  |  |  |  |  |  |  |  |  |  |  |  |  |  |  |  |  |  |  |  |
| **Originality** |  | Spearman's rho |  | 0.586 | *** | 0.493 | *** | — |  |  |  |  |  |  |  |  |  |  |  |  |  |  |  |  |  |  |  |  |
|  |  | p-value |  | < .001 |  | < .001 |  | — |  |  |  |  |  |  |  |  |  |  |  |  |  |  |  |  |  |  |  |  |
| **Flexibility** |  | Spearman's rho |  | 0.793 | *** | 0.746 | *** | 0.477 | *** | — |  |  |  |  |  |  |  |  |  |  |  |  |  |  |  |  |  |  |
|  |  | p-value |  | < .001 |  | < .001 |  | < .001 |  | — |  |  |  |  |  |  |  |  |  |  |  |  |  |  |  |  |  |  |
| **Elaboration** |  | Spearman's rho |  | 0.731 | *** | 0.433 | *** | 0.085 |  | 0.390 | *** | — |  |  |  |  |  |  |  |  |  |  |  |  |  |  |  |  |
|  |  | p-value |  | < .001 |  | < .001 |  | 0.455 |  | < .001 |  | — |  |  |  |  |  |  |  |  |  |  |  |  |  |  |  |  |
| **Total Gestures** |  | Spearman's rho |  | -0.028 |  | -0.060 |  | -0.178 |  | -0.229 | * | 0.238 | * | — |  |  |  |  |  |  |  |  |  |  |  |  |  |  |
|  |  | p-value |  | 0.805 |  | 0.594 |  | 0.113 |  | 0.041 |  | 0.033 |  | — |  |  |  |  |  |  |  |  |  |  |  |  |  |  |
| **Beat** |  | Spearman's rho |  | -0.095 |  | -0.167 |  | -0.064 |  | -0.179 |  | 0.055 |  | 0.451 | *** | — |  |  |  |  |  |  |  |  |  |  |  |  |
|  |  | p-value |  | 0.402 |  | 0.140 |  | 0.575 |  | 0.112 |  | 0.627 |  | < .001 |  | — |  |  |  |  |  |  |  |  |  |  |  |  |
| **Iconic** |  | Spearman's rho |  | 0.097 |  | 0.060 |  | -0.114 |  | -0.117 |  | 0.329 | ** | 0.750 | *** | -0.024 |  | — |  |  |  |  |  |  |  |  |  |  |
|  |  | p-value |  | 0.392 |  | 0.599 |  | 0.316 |  | 0.300 |  | 0.003 |  | < .001 |  | 0.833 |  | — |  |  |  |  |  |  |  |  |  |  |
| **Metaphoric** |  | Spearman's rho |  | 0.115 |  | 0.055 |  | -0.055 |  | 0.004 |  | 0.295 | ** | 0.437 | *** | 0.210 |  | 0.196 |  | — |  |  |  |  |  |  |  |  |
|  |  | p-value |  | 0.309 |  | 0.626 |  | 0.628 |  | 0.972 |  | 0.008 |  | < .001 |  | 0.062 |  | 0.082 |  | — |  |  |  |  |  |  |  |  |
| **Deictic** |  | Spearman's rho |  | -0.066 |  | -0.052 |  | -0.175 |  | -0.083 |  | 0.040 |  | 0.295 | ** | 0.065 |  | 0.125 |  | 0.087 |  | — |  |  |  |  |  |  |
|  |  | p-value |  | 0.562 |  | 0.647 |  | 0.121 |  | 0.466 |  | 0.724 |  | 0.008 |  | 0.565 |  | 0.270 |  | 0.445 |  | — |  |  |  |  |  |  |
| **Palm-revealing** |  | Spearman's rho |  | 0.060 |  | 0.013 |  | 0.049 |  | 0.085 |  | 0.029 |  | 0.384 | *** | 0.052 |  | 0.083 |  | 0.359 | ** | 0.080 |  | — |  |  |  |  |
|  |  | p-value |  | 0.595 |  | 0.912 |  | 0.666 |  | 0.453 |  | 0.802 |  | < .001 |  | 0.649 |  | 0.465 |  | 0.001 |  | 0.479 |  | — |  |  |  |  |
| **MIT** |  | Spearman's rho |  | -0.074 |  | -0.157 |  | -0.003 |  | -0.011 |  | -0.017 |  | 0.017 |  | 0.061 |  | 0.018 |  | -0.010 |  | 0.069 |  | -0.040 |  | — |  |  |
|  |  | p-value |  | 0.513 |  | 0.165 |  | 0.982 |  | 0.923 |  | 0.881 |  | 0.880 |  | 0.589 |  | 0.877 |  | 0.932 |  | 0.545 |  | 0.722 |  | — |  |  |
| Note. * p < .05, ** p < .01, *** p < .001 | | | | | | | | | | | | | | | | | | | | | | | | | | | | |
|  | | | | | | | | | | | | | | | | | | | | | | | | | | | | |

**Table S2.** Spearman Correlational Matrix for Group 1 gesture-spontaneous condition

**Table S3.** Spearman Correlational Matrix for Group 1 gesture-encouraged condition

|  | | |  | | **AUT** | | **Fluency** | | **Originality** | | **Flexibility** | | **Elaboration** | | **Total Gestures** | | | **Beat** | | | **Iconic** | | | **Metaphoric** | | | **Deictic** | | | **Palm-revealing** | | | **MIT** | | |  |  |
| --- | --- | --- | --- | --- | --- | --- | --- | --- | --- | --- | --- | --- | --- | --- | --- | --- | --- | --- | --- | --- | --- | --- | --- | --- | --- | --- | --- | --- | --- | --- | --- | --- | --- | --- | --- | --- | --- |
| **AUT** | |  | Spearman's rho |  | — |  |  |  |  |  |  |  |  |  |  |  |  | |  |  | |  |  | |  |  | |  |  | |  |  | |  |  | |  |
|  | |  | p-value |  | — |  |  |  |  |  |  |  |  |  |  |  |  | |  |  | |  |  | |  |  | |  |  | |  |  | |  |  | |  |
| **Fluency** | |  | Spearman's rho |  | 0.682 | *** | — |  |  |  |  |  |  |  |  |  |  | |  |  | |  |  | |  |  | |  |  | |  |  | |  |  | |  |
|  | |  | p-value |  | < .001 |  | — |  |  |  |  |  |  |  |  |  |  | |  |  | |  |  | |  |  | |  |  | |  |  | |  |  | |  |
| **Originality** | |  | Spearman's rho |  | 0.717 | *** | 0.412 | *** | — |  |  |  |  |  |  |  |  | |  |  | |  |  | |  |  | |  |  | |  |  | |  |  | |  |
|  | |  | p-value |  | < .001 |  | < .001 |  | — |  |  |  |  |  |  |  |  | |  |  | |  |  | |  |  | |  |  | |  |  | |  |  | |  |
| **Flexibility** | |  | Spearman's rho |  | 0.548 | *** | 0.508 | *** | 0.264 | * | — |  |  |  |  |  |  | |  |  | |  |  | |  |  | |  |  | |  |  | |  |  | |  |
|  | |  | p-value |  | < .001 |  | < .001 |  | 0.018 |  | — |  |  |  |  |  |  | |  |  | |  |  | |  |  | |  |  | |  |  | |  |  | |  |
| **Elaboration** | |  | Spearman's rho |  | 0.840 | *** | 0.333 | ** | 0.453 | *** | 0.296 | ** | — |  |  |  |  | |  |  | |  |  | |  |  | |  |  | |  |  | |  |  | |  |
|  | |  | p-value |  | < .001 |  | 0.003 |  | < .001 |  | 0.008 |  | — |  |  |  |  | |  |  | |  |  | |  |  | |  |  | |  |  | |  |  | |  |
| **Total Gestures** | |  | Spearman's rho |  | 0.231 | * | 0.313 | ** | 0.224 | * | 0.164 |  | 0.087 |  | — |  |  | |  |  | |  |  | |  |  | |  |  | |  |  | |  |  | |  |
|  | |  | p-value |  | 0.039 |  | 0.005 |  | 0.046 |  | 0.147 |  | 0.440 |  | — |  |  | |  |  | |  |  | |  |  | |  |  | |  |  | |  |  | |  |
| **Beat** | |  | Spearman's rho |  | 0.017 |  | -0.046 |  | 0.009 |  | -0.025 |  | 0.008 |  | 0.236 | * | — | |  |  | |  |  | |  |  | |  |  | |  |  | |  |  | |  |
|  | |  | p-value |  | 0.883 |  | 0.686 |  | 0.939 |  | 0.826 |  | 0.941 |  | 0.035 |  | — | |  |  | |  |  | |  |  | |  |  | |  |  | |  |  | |  |
| **Iconic** | |  | Spearman's rho |  | 0.243 | * | 0.312 | ** | 0.277 | * | 0.157 |  | 0.104 |  | 0.772 | *** | -0.251 | | * | — | |  |  | |  |  | |  |  | |  |  | |  |  | |  |
|  | |  | p-value |  | 0.030 |  | 0.005 |  | 0.013 |  | 0.165 |  | 0.359 |  | < .001 |  | 0.025 | |  | — | |  |  | |  |  | |  |  | |  |  | |  |  | |  |
| **Metaphoric** | |  | Spearman's rho |  | 0.416 | *** | 0.292 | ** | 0.178 |  | 0.178 |  | 0.424 | *** | 0.261 | * | 0.056 | |  | 0.105 | |  | — | |  |  | |  |  | |  |  | |  |  | |  |
|  | |  | p-value |  | < .001 |  | 0.009 |  | 0.115 |  | 0.114 |  | < .001 |  | 0.020 |  | 0.623 | |  | 0.356 | |  | — | |  |  | |  |  | |  |  | |  |  | |  |
| **Deictic** | |  | Spearman's rho |  | 0.178 |  | 0.246 | * | 0.053 |  | 0.286 | * | 0.068 |  | 0.224 | * | -0.041 | |  | 0.085 | |  | 0.124 | |  | — | |  |  | |  |  | |  |  | |  |
|  | |  | p-value |  | 0.114 |  | 0.028 |  | 0.638 |  | 0.010 |  | 0.546 |  | 0.046 |  | 0.721 | |  | 0.456 | |  | 0.271 | |  | — | |  |  | |  |  | |  |  | |  |
| **Palm-revealing** | |  | Spearman's rho |  | 0.113 |  | 0.193 |  | 0.115 |  | 0.162 |  | 0.065 |  | 0.359 | ** | 0.047 | |  | 0.136 | |  | 0.080 | |  | 0.185 | |  | — | |  |  | |  |  | |  |
|  | |  | p-value |  | 0.318 |  | 0.086 |  | 0.308 |  | 0.151 |  | 0.564 |  | 0.001 |  | 0.679 | |  | 0.230 | |  | 0.478 | |  | 0.101 | |  | — | |  |  | |  |  | |  |
| **MIT** | |  | Spearman's rho |  | -0.060 |  | -0.123 |  | -0.046 |  | 0.026 |  | 0.018 |  | -0.144 |  | -0.115 | |  | -0.074 | |  | -0.289 | | ** | -0.075 | |  | 0.188 | |  | — | |  |  | |  |
|  | |  | p-value |  | 0.597 |  | 0.276 |  | 0.684 |  | 0.818 |  | 0.877 |  | 0.203 |  | 0.309 | |  | 0.516 | |  | 0.009 | |  | 0.509 | |  | 0.095 | |  | — | |  |  | |  |
|  |  |  |  |  |  |  |  |  |  |  |  |  |  |  |  |  |  |  |  |  |  |  |  |  |  |  |  |  |  |  |  |  |  |  |  |  |  |
|  |  |  |  |  |  |  |  |  |  |  |  |  |  |  |  |  |  |  |  |  |  |  |  |  |  |  |  |  |  |  |  |  |  |  |  |  |  |
|  |  |  |  |  |  |  |  |  |  |  |  |  |  |  |  |  |  |  |  |  |  |  |  |  |  |  |  |  |  |  |  |  |  |  |  |  |  |
| Note. * p < .05, ** p < .01, *** p < .001 | | | | | | | | | | | | | | | | | | | | | | | | | | | | | | | | | | | | | |
|  | | | | | | | | | | | | | | | | | | | | | | | | | | | | | | | | | | | | | |

**Table S4.** Spearman Correlational Matrix for Group 2 (gesture-encouraged condition)

|  | |  | | **AUT** | | **Fluency** | | **Originality** | | **Flexibility** | | **Elaboration** | | **Total Gestures** | | | **Beat** | | | **Iconic** | | | **Metaphoric** | | | **Deictic** | | | **Palm-revealing** | | | **MIT** | | |  |
| --- | --- | --- | --- | --- | --- | --- | --- | --- | --- | --- | --- | --- | --- | --- | --- | --- | --- | --- | --- | --- | --- | --- | --- | --- | --- | --- | --- | --- | --- | --- | --- | --- | --- | --- | --- |
| **AUT** |  | Spearman's rho |  | — |  |  |  |  |  |  |  |  |  |  |  |  | |  |  | |  |  | |  |  | |  |  | |  |  | |  |  |  |
|  |  | p-value |  | — |  |  |  |  |  |  |  |  |  |  |  |  | |  |  | |  |  | |  |  | |  |  | |  |  | |  |  |  |
| **Fluency** |  | Spearman's rho |  | 0.861 | *** | — |  |  |  |  |  |  |  |  |  |  | |  |  | |  |  | |  |  | |  |  | |  |  | |  |  |  |
|  |  | p-value |  | < .001 |  | — |  |  |  |  |  |  |  |  |  |  | |  |  | |  |  | |  |  | |  |  | |  |  | |  |  |  |
| **Originality** |  | Spearman's rho |  | 0.727 | *** | 0.588 | *** | — |  |  |  |  |  |  |  |  | |  |  | |  |  | |  |  | |  |  | |  |  | |  |  |  |
|  |  | p-value |  | < .001 |  | < .001 |  | — |  |  |  |  |  |  |  |  | |  |  | |  |  | |  |  | |  |  | |  |  | |  |  |  |
| **Flexibility** |  | Spearman's rho |  | 0.793 | *** | 0.691 | *** | 0.524 | *** | — |  |  |  |  |  |  | |  |  | |  |  | |  |  | |  |  | |  |  | |  |  |  |
|  |  | p-value |  | < .001 |  | < .001 |  | < .001 |  | — |  |  |  |  |  |  | |  |  | |  |  | |  |  | |  |  | |  |  | |  |  |  |
| **Elaboration** |  | Spearman's rho |  | 0.794 | *** | 0.527 | *** | 0.349 | ** | 0.491 | *** | — |  |  |  |  | |  |  | |  |  | |  |  | |  |  | |  |  | |  |  |  |
|  |  | p-value |  | < .001 |  | < .001 |  | 0.002 |  | < .001 |  | — |  |  |  |  | |  |  | |  |  | |  |  | |  |  | |  |  | |  |  |  |
| **Total Gestures** |  | Spearman's rho |  | -0.033 |  | -0.144 |  | 0.014 |  | -0.191 |  | 0.092 |  | — |  |  | |  |  | |  |  | |  |  | |  |  | |  |  | |  |  |  |
|  |  | p-value |  | 0.772 |  | 0.202 |  | 0.902 |  | 0.090 |  | 0.419 |  | — |  |  | |  |  | |  |  | |  |  | |  |  | |  |  | |  |  |  |
| **Beat** |  | Spearman's rho |  | -0.031 |  | -0.156 |  | -0.012 |  | -0.141 |  | 0.064 |  | 0.470 | *** | — | |  |  | |  |  | |  |  | |  |  | |  |  | |  |  |  |
|  |  | p-value |  | 0.788 |  | 0.167 |  | 0.914 |  | 0.214 |  | 0.573 |  | < .001 |  | — | |  |  | |  |  | |  |  | |  |  | |  |  | |  |  |  |
| **Iconic** |  | Spearman's rho |  | 0.253 | * | 0.195 |  | 0.148 |  | 0.116 |  | 0.300 | ** | 0.507 | *** | -0.228 | | * | — | |  |  | |  |  | |  |  | |  |  | |  |  |  |
|  |  | p-value |  | 0.023 |  | 0.083 |  | 0.190 |  | 0.305 |  | 0.007 |  | < .001 |  | 0.042 | |  | — | |  |  | |  |  | |  |  | |  |  | |  |  |  |
| **Metaphoric** |  | Spearman's rho |  | 0.186 |  | 0.046 |  | 0.158 |  | 0.150 |  | 0.194 |  | 0.258 | * | 0.231 | | * | -0.026 | |  | — | |  |  | |  |  | |  |  | |  |  |  |
|  |  | p-value |  | 0.098 |  | 0.687 |  | 0.162 |  | 0.183 |  | 0.085 |  | 0.021 |  | 0.039 | |  | 0.822 | |  | — | |  |  | |  |  | |  |  | |  |  |  |
| **Deictic** |  | Spearman's rho |  | 0.066 |  | 0.057 |  | 0.052 |  | -0.069 |  | 0.080 |  | 0.377 | *** | 0.393 | | *** | -0.091 | |  | 0.190 | |  | — | |  |  | |  |  | |  |  |  |
|  |  | p-value |  | 0.563 |  | 0.616 |  | 0.646 |  | 0.542 |  | 0.483 |  | < .001 |  | < .001 | |  | 0.422 | |  | 0.092 | |  | — | |  |  | |  |  | |  |  |  |
| **Palm-revealing** |  | Spearman's rho |  | -0.200 |  | -0.109 |  | -0.102 |  | -0.295 | ** | -0.114 |  | 0.198 |  | -0.124 | |  | 0.083 | |  | -0.158 | |  | -0.131 | |  | — | |  |  | |  |  |  |
|  |  | p-value |  | 0.076 |  | 0.335 |  | 0.370 |  | 0.008 |  | 0.315 |  | 0.078 |  | 0.272 | |  | 0.465 | |  | 0.162 | |  | 0.246 | |  | — | |  |  | |  |  |  |
| **MIT** |  | Spearman's rho |  | 0.058 |  | 0.074 |  | -0.051 |  | 0.110 |  | 0.080 |  | 0.126 |  | 0.056 | |  | 0.088 | |  | 0.038 | |  | 0.151 | |  | 0.014 | |  | — | |  |  |  |
|  |  | p-value |  | 0.610 |  | 0.516 |  | 0.653 |  | 0.333 |  | 0.482 |  | 0.266 |  | 0.619 | |  | 0.438 | |  | 0.737 | |  | 0.182 | |  | 0.903 | |  | — | |  |  |  |
| Note. * p < .05, ** p < .01, *** p < .001 | | | | | | | | | | | | | | | | | | | | | | | | | | | | | | | | | | | |
|  | | | | | | | | | | | | | | | | | | | | | | | | | | | | | | | | | | | |
